# “I am sick, but that’s not all that I am”: patient perspectives on psychological adaptation over time to inborn errors of immunity

**DOI:** 10.1007/s12687-024-00758-z

**Published:** 2025-01-06

**Authors:** Breanna J. Beers, Hannah R. Davidson-Swinton, Katie L. Lewis, Michael R. Setzer, Magdalena A. Walkiewicz, Morgan N. Similuk

**Affiliations:** 1 Centralized Sequencing Program, National Institute of Allergy and Infectious Diseases (NIAID), Bethesda, MD USA; 2 Department of Health, Behavior, and Society, Johns Hopkins Bloomberg School of Public Health, Baltimore, MD USA; 3 Telomere Center, Johns Hopkins University School of Medicine, Baltimore, MD USA; 4 Department of Genetic Medicine, Johns Hopkins University School of Medicine, Baltimore, MD USA; 5 Department of Medical and Clinical Psychology, Uniformed Services University of the Health Sciences, Bethesda, MD USA

**Keywords:** Adaptation, Adjustment, Immunodeficiency, Rare, Chronic, Qualitative

## Abstract

Inborn errors of immunity (IEI) are rare heritable disorders of the immune system predisposing to atypical infections, autoimmunity, inflammation, and risk of malignancy. Adaptation is the process of incorporating stressful experiences into one’s life; these experiences may include onset of illness, receiving a diagnosis, or suffering without a diagnosis. Poor adaptation is linked to adverse outcomes including psychiatric comorbidities and decreased well-being. Most people with chronic illnesses and rare diseases adapt to their condition with time, but little is known about how this happens. We seek to address this gap in knowledge by investigating features of successful adaptation to IEI over time to help providers facilitate this process among those who struggle. We interviewed 20 self-reportedly well-adapted adults with IEI about their experiences of adaptation. We identified three overarching themes. First, many participants grappled with shame and loss but ultimately integrated their illness into their lives through acceptance, gratitude, and self-compassion. Second, knowledge and choice contributed to participants’ perceived control, but most participants were also forced to acknowledge the limits of that control when confronting unpredictable symptoms. Finally, social relationships could influence adaptation either positively or negatively. Notably, many participants found meaning in giving back to their communities, especially by educating others about their condition. Ultimately, participants showed that adaptation to life with IEI is complex and ongoing, requiring frequent recalibration to emerging challenges in new life stages. We provide recommendations to providers to help the results of this study shape their discussions with patients.

**Supplementary Information:**

The online version contains supplementary material available at 10.1007/s12687-024-00758-z.

## Introduction

Psychological adaptation is the mental and emotional processes through which individuals adjust to and manage the impact of stressful life events. Such events can include receiving a clinical diagnosis, receiving a genetic explanation for clinical illness, or facing ongoing symptoms in the absence of a clearly defined diagnosis (Taylor 1983; Setzer et al. 2023).

Like many other rare, chronic, and often life-disrupting conditions, living with an inborn error of immunity (IEI) requires adjusting to new limitations and routines while coping with significant uncertainty. IEIs include nearly 500 distinct heritable disorders manifesting with atypical infections, autoimmunity, inflammation, and risk of malignancy. Severity varies widely even within families but affected individuals can face multiple hospitalizations and lifelong treatments (Hsueh et al. 2024). Even between acute infections, symptoms can fluctuate from day to day. Many IEI patients experience extended diagnostic delay, which can detrimentally impact treatment and leave patients without a clear path forward (Anderson et al. 2022; Bahrami et al. 2020; Branch et al. 2021; Immune Deficiency Foundation 2023; Slade et al. 2018; Urschel et al. 2009). Even with comprehensive genomic evaluation, yield of genetic testing is around 30–40%, leaving most patients with clinically suspected IEI without a molecular diagnosis (Similuk et al. 2022; Vorsteveld et al. 2021). Patients face difficult decisions around management, transplant, and family planning, often with limited prognostic information.

Theoretical frameworks conceptualize how individuals make sense of threatening experiences, offering structured insights into the underlying processes. Shelley Taylor’s theory of psychological adaptation, based on interviews with breast cancer survivors, organizes adaptation into three tasks: (1) reestablishing self-esteem after the damage caused by the event; (2) finding meaning in the experience, often by seeking causal attributions; and (3) regaining mastery over one’s life in the wake of the event (Taylor 1983). Steven Lepore’s social-cognitive processing model further suggests the process of adaptation is moderated by others’ responses to sharing one’s experiences. “Supportive, receptive, or noncritical” responses facilitate adaptation; “unsupportive, unreceptive, or critical” responses can be barriers to it (Lepore 2001). Importantly, poor adaptation is correlated with a number of adverse outcomes, including anxiety, depression, caregiver overload, and decreased well-being (Rodríguez et al. 2024; Tao et al. 2023; Heyink 1993; Biesecker et al. 2013).

Studies of adaptation reflect that the process of adaptation varies widely among individuals. The patients who struggle most to adjust may benefit from additional interventions (Biesecker and Erby 2008). Among patients with IEIs, studies have found decreased quality of life (Barlogis et al. 2017; Battersby et al. 2019; Nicholson et al. 2022; Titman et al. 2014; Xiao et al. 2024) and increased psychiatric comorbidities (Manusama et al. 2022; Ocakoglu et al. 2018). Psychosocial factors, most prominently anxiety and social maladjustment, have been shown to play an important role in determining these outcomes independent of symptom severity and may be strategic targets for intervention (Cohen and Biesecker 2010; Similuk et al. 2016; Gumusburun et al. 2024; Michniacki et al. 2019; Zhang et al. 2020; Ahmed Meelad et al. 2022). One recent survey study found that perceived control is significantly associated with psychological adaptation in participants with IEI, with and without genetic diagnoses (Setzer et al. 2023). However, there is only limited research on other facets of how people with IEI positively adapt to life with a rare, chronic, unpredictable illness, particularly through a qualitative lens (Similuk et al. 2016; Peshko et al. 2019). We aim to address this knowledge gap by investigating features of successful adaptation to IEI over time and providing new insights that will equip providers to identify and help those who struggle.

## Methods

Participants were recruited from the National Institute of Allergy and Infectious Diseases (NIAID) Centralized Sequencing Program (CSP) and participated in a prior survey study. In brief, the survey study recruited adults with IEI from 2017 to 2021 and included questions covering constructs including perceived control, mental health, and adaptation (Setzer et al. 2023). To be eligible for the present interview study, participants must have been 18 – 40 years old, reported above-average adaptation compared to other survey participants on the Psychological Adaptation Scale (PAS), and indicated scores consistent with at least mild anxiety and/or depression on the PROMIS 29 Profile v2.1 (Biesecker et al. 2013; Choi et al. 2014; Schalet et al. 2014). Recruitment continued until meaning saturation was achieved; ultimately, 35 eligible participants were invited up to three times via email. The semi-structured interview guide included questions about the process of adaptation over time, as well as meaning making and mastery (Online Resource 1).

Two researchers performed interviews remotely from June through October 2022. Interviews were recorded and transcribed. Identifying information was removed. The primary coder (BJB) and a supervisor (KLL) reviewed and independently coded three transcripts before developing a codebook. The primary coder then coded all transcripts and met with the supervisor to discuss questions and challenging passages. Coded data were analyzed using an inductive, semantic thematic approach (Braun and Clarke 2006). Inductive approaches generate codebooks and themes from interview data rather than an external framework; semantic analysis focuses on participants’ explicit descriptions of their experiences rather than implicit concepts. Findings were summarized and sent to participants for feedback by email and phone.

Written informed consent was obtained for all participants and the study was approved by the National Institutes of Health (NIH) Institutional Review Board (NCT03206099).

### Positionality

This project and its analysis were executed by all members of the authorship team, the majority of whom are female and one of whom is male. Several members of the authorship team work as, or were in training to be, genetic counselors at the time of data collection and/or writing this paper. Three of these genetic counselors had/have close working experiences within the IEI community through the CSP, which included previously consenting several of the participants to genome sequencing.

## Results

### Participant characteristics

Overall, 20/35 participants (57%) agreed to participate. Median interview length was 55 min (range: 32—98 min). By self-report, the interview cohort was predominantly white (17/20, 85%), non-Hispanic (14/20, 70%), female (13/20, 65%), and had been living with IEI for over ten years prior to the initial survey (14/20, 70%). The most common self-reported diagnoses were common variable immune deficiency (5/20, 20%) and GATA2 deficiency (5/20, 20%). Seven participants (35%) had undergone hematopoietic stem cell transplant (HCST) prior to the interview. Participant characteristics are described in Table 1.

**Table 1:** Participant characteristics

	**Attribute**	**N**	**%**	**Mean**	**St. Dev**
**Age**	Years	-	-	32.3	6.51
**Sex**	Female	13	65%	-	-
	Male	7	35%	-	-
**Race**	Asian	1	5%	-	-
	Native Hawaiian or Pacific Islander	1	5%	-	-
	White	17	85%	-	-
	Unknown	1	5%	-	-
**Ethnicity**	Hispanic or Latino	5	25%	-	-
	Not Hispanic or Latino	14	70%	-	-
	Unknown	1	5%	-	-
**Self-reported diagnoses**	Common variable immune deficiency (CVID)	5	25%	-	-
	GATA2 deficiency	5	25%	-	-
	DOCK8-related hyper-IgE syndrome, Job’s syndrome	2	10%	-	-
	Autoimmune polyendocrinopathy-candidiasis-ectodermal dystrophy (APECED)	1	5%	-	-
	Chronic granulomatous disease (CGD)	1	5%	-	-
	Primary ciliary deficiency (PCD)	1	5%	-	-
	Other	5	25%	-	-
**HSCT**	Yes	7	35%	-	-
	No	13	65%	-	-
**Time since illness onset**	< 1 year	1	5%	-	-
	1—5 years	1	5%	-	-
	5—10 years	3	15%	-	-
	> 10 years	14	70%	-	-
	Unsure	1	5%	-	-
**Adaptation**	PAS score	-	-	4.16	0.33
**Anxiety**	PROMIS 29 Profile v2.1	-	-	61.7	7.71
**Depression**	PROMIS 29 Profile v2.1	-	-	54.2	7.54

### Thematic analysis

Through inductive, semantic thematic analysis, we identified three major themes: rewriting narratives, navigating disruptions, and renegotiating community.

### Theme 1: Rewriting narratives

#### Subtheme 1: from resistance to acceptance

Participants initially resisted their illness before gradually accepting it. Participants connected the concept of resistance to focusing on where they felt they “should” be, driven by comparison to others or internalized shame, as shown in Table 2. Four participants (20%) explicitly used the word “shame” to characterize their relationship with their illness; many more implicitly described experiencing it. Notably, shame sprung from a variety of underlying causes and contributed to self-reinforcing behaviors, as shown in Table 3.

**Table 2:** Rewriting the narrative from resistance to acceptance

RESISTANCE	ACCEPTANCE	ID
***Shame***	***Self-compassion***	
“I buried it in a way that I didn’t let myself feel it.”	“I have other things that I’m going through than my friends, but that doesn’t make me a broken person.”	37 F, S4328287
“I did a really good job at hiding for a really long time…I was just trying to fit in.”	“It feels so much better to come out and just be myself.”	37 F, S4328287
“I would push through it, but then it would make it worse for days.”	“I’ve learned to rest when I feel exhausted.”	38 F, S9033160
***Comparison***	***Gratitude***	
“If you are only busy with your illness, it will make you a depressive person.”	“But if you try to live your life at a positive way, it will give your life more joy and more rest.”	39 F, S0583046
“Of course, there are days that I'm asking myself why I feel the pain, why I'm getting to all of this. Why is all of this happening?”	“But then I'm going to listen to music and do meditation and look outside and think about how beautiful life is.”	39 F, S0583046
“Your thoughts, too, can make you sick.”	“So think positive. … I’m getting ready to be a new person.”	37 M, S8251256
**DEFINING**	**INTEGRATING**	
“It’s just become such a huge part of my life.”	“I need to stop letting this illness define who I am, because it’s not who I am.”	27 F, S7235706
“To be able to manage it on my own and not be defined by my health.”	“Instead, to learn from it and help other people go through it as well.”	37 F, S4328287
“I might be sick and I might get admitted to the hospital every now and again. Yeah, I've had a lot of surgeries and those surgeries have left scars and they've left this and that.”	“It was kind of this realization that nothing’s going to stop me. … At the end of the day, that's not all of who I am. I can be so much more than the person that was just a victim to an illness.”	23 M, S3173363

**Table 3:** Adaptation in the context of shame, a key barrier to acceptance

Drivers of shame	Lack of agency	“I almost hated myself for that, too, because I never knew what was wrong with me; I always would almost blame myself.” *–25 F, S6632797*
	Feeling different	“I just wanted so badly to fit in and look like my friends and not behind the scenes deal with this stuff that affected me, that I didn’t realize it was affecting me then emotionally.” *–37 F, S4328287*
	Survivorship	“I had met somebody in the hospital…I watched him die in the ICU.” *–25 F, S6632797*
	Burden of care	“I am dependent. I need him. Sometimes it’s shameful.” *–39 F, S0583046*
	Heritability of disease	“Sometimes I blame myself that [my daughter] has [GATA2].” *–39 F, S0583046*
Maladaptive responses to shame	Suppress emotions	“I buried it in a way that I didn’t let myself feel it.” *–37 F, S4328287*
	Avoid discussing illness	“I did a really good job at hiding for a really long time…I was just trying to fit in.” *–37 F, S4328287*
	Resist limitations of illness	“I would push through it, but then it would make it worse for days.” *–38 F, S9033160*
	Ignore health demands	“I was looking to put Band-Aids on everything physically and emotionally.” *–37 F, S4328287*
Adaptive responses to shame	Reframe internal narrative	“When I started telling myself a different story, it changed everything.” *–39 F, S2644543*
	Acknowledge illness to others	“There are certain times where … I’m not in the mood to talk, and then when I talk, I’m like, “I’m so glad I talked. I’m so glad someone listened. I’m so glad someone was there for me.”” *–37 F, S4328287*
	Accept ongoing reality of illness	“There’s nothing that’s going to change it.” *–25 F, S6632797*
	Internalize uncaused nature of illness	“It wasn’t my fault; it is what it is. People get sick and that’s just the reality of life.” *–40 F, S5228470*
	Give oneself permission to alter lifestyle	“That was something I had to teach myself, was that it’s okay to rest.” *–25 F, S6561773*

If resistance was characterized by (unsuccessful) attempts to ignore physical and emotional needs, acceptance meant acknowledging the reality of illness without being crushed by the weight of that reality. Some overcame shame through self-compassion, which enabled them to open up about their experiences to others, reframe their responsibility for their illness, and forgive themselves for their own limitations: “not being hard on yourself if you feel like you can’t do something” (40 F, S5228470). For instance, one participant situated her illness in the context of shared human struggles, which helped her feel less ashamed of her particular burden:

“There’s no reason as to why things happen to certain people and not to others, so there's no point in thinking my life should be different. … This is my thing, the mountain I'm climbing, and everyone has their own.” *–39 F, S2644543*

As a result, she said, she developed greater “self-awareness and humility and acceptance,” seeing herself as “both completely insignificant and of incalculable significance.” She continued, “I get to decide how I’m going to respond to the things that have happened to me” (39 F, S2644543). For this participant, living with IEI created an opportunity to assert control over her emotional world even in uncontrollable circumstances.

Similarly, another participant said the unpredictable nature of chronic illness presented her with a choice of focusing on healthy days or hard ones:

“When you have chronic illness, you don’t take moments for granted as much because some days are really good and some days are really bad. So you learn to capitalize on the good days and to appreciate the good days, and also just not get rattled as much by little things that don’t matter.” *–28 F, S6531719*

Choosing to be grateful for the good days helped this participant move through the bad ones. She also shared that practicing her religion helped cultivate this perspective. Other participants likewise cited behaviors that fostered gratitude, including meditating, writing, going outdoors, listening to music, pursuing hobbies, and seeking therapy. As a result, several participants grew through coping with their illness. For instance, one participant identified her increased ability to live in the moment:

“Realizing that I wasn't going to go back to being the old [me]…Actually, I feel like I'm happier overall now, even though I have all this anxiety and depression. ... I can just stare at a leaf and be like, ‘Wow, look at that leaf,’ and find the beauty in it, where before I had to always be moving. So I think finding the beauty in the little things, and I feel like I've grasped a part of, like, the meaning of life, more than I did before.” *–38 F, S9033160*

For her, like many participants, acceptance involved both acknowledging the ongoing reality of her illness and embracing the person she became as a result of her illness.

#### Subtheme 2: from defining to integrating

Many participants grappled with and ultimately rejected the concept of illness as identity, as shown in Table 2. They said “[My illness is] something that I deal with every day, but it’s not who I am” (28 F, S6531719) and emphasized that they were “not letting my condition or disease state control my life” (39 F, S6509727). In fact, defining oneself apart from IEI was closely tied to empowerment:

“Nothing’s going to stop me. I might be sick, and I might get admitted to the hospital every now and again. Yeah, I've had a lot of surgeries and those surgeries have left scars. ... But at the end of the day, that's not all of who I am. I can be so much more than the person that was just a victim to an illness.” *–23 M, S3173363*

For this participant, a narrative which solely focused on his medical odyssey would not be a complete representation of his story. Instead, participants said, they had to learn how to integrate their illness into their lives without letting it define their identity. This is an especially challenging feat in IEI, which often involves recurrent infections, frequent hospital visits, and daily risk management. To that end, some were initially overwhelmed by medical trauma and struggled to restart their lives as their symptoms stabilized; others were so resistant to being labeled chronically ill that they tried to compartmentalize their illness and carry on as “normal.” Ultimately, to adapt, both groups had to learn to incorporate their illness into their lives—frequent doctor’s visits, lifestyle alterations, emotional support—without being consumed by it. As one participant said, “Yes, I am sick. … but that’s not all that I am. … That’s a part of my life, but it’s not my entire life. So I can make a life beyond a hospital bed” (23 M, S3173363).

#### Subtheme 3: an ongoing process

Even among a cohort of relatively well-adapted participants, several explicitly stated that they are still undergoing a continual process of adaptation. One participant described her illness this way: “It’s like your whole entire life’s been hit by a train – and it just keeps coming back. It doesn’t keep rolling down the tracks. It just keeps making circles and knocking you off your feet” (27 F, S8556521).

When participants reflected back on their illness experiences, they identified discrete challenges that emerged in various life stages: elementary school worries were different from adolescent insecurities; dating difficulties in young adulthood were distinct to grieving infertility secondary to transplant. While some coping skills were transferable, new challenges also required novel approaches to adaptation. As one participant said, “That process [of accepting one’s illness] is very messy and circuitous and it’s like one step forward, two steps back, feeling around in the dark… And it’s still happening” (39 F, S2644543). Crucially, the ongoing, dynamic nature of the adaptation process highlights the role of not only time, but effort, context, and support.

### Theme 2: Navigating disruptions

#### Subtheme 1: control what you can and accept what you can’t

Nearly every participant sought to control what they could about their illness and their lives. However, given the unpredictable and chronic nature of IEI, participants had to accept what was outside their control. This delicate balance extended beyond the medical domain to touch on participants’ personal lives and social circles; examples of this tension are given in Table 4.

**Table 4:** Balancing control and acceptance in medical, personal, and social spheres

	Control what you can		Accept what you can’t	
Medically	Learn about illness	“If you know more about your illness, you can live your life better. … If you don't know much about your illness, you keep struggling and keep asking, ‘Why is this happening this to me? What is happening? Why do I get all those infections?’” *–39 F, S0583046*	Adjust to new physical limitations	“I couldn’t do normal things. … Finding different programs or different exercises that I can do … has helped a lot. I think I found a good balance of knowing what I can and can’t do and doing what I can to make it so maybe someday I can do those things that I now can’t do.” *–27 F, S7235706*
	Develop body awareness	“Something coming on or something not feeling right, I’m very attuned to that. I think I’ve become a pro at my own body.” *–37 F, S4328287*	Understand the limits of non-expert providers	“Even if a doctor tells me something is not true … I don’t feel … like I’m crazy, because I know my body better than a doctor could.” *–25 F, S6561773*
	Gain fluency with healthcare system	“I’ve also learned a lot of the medical terminology for my issues; that makes me feel a little bit more in control whenever I’m talking to a doctor.” *–25 F, S6561773*	Accept diagnostic and prognostic uncertainty	“What I have still is just kind of like unknown, and I think that's what's the hardest part. … I've just accepted that whether it's got some acronym or not, it doesn't really matter.” *–38 F, S9033160*
	Manage infectious exposures	“I can control, for the most part, what germs I've been exposed to, who I'm exposed to who's sick, if I'm exposed to anyone that's sick.” *–23 M, S3173363*	Recognize inevitability of exposure	“It’s a matter of preventing the germs and still living my life, rather than letting the prevention of germs rule my life.” *–23 M, S3173363*
Personally	Proactively manage mental health	“Diving into therapy and learning how to manage stress and all the self-care aspects for sure so I wouldn't spiral and get sick. … We need to figure out what I need to do to help myself.” *–40 F, S2026162*	Recognize that mental and physical health interrelate	“Everybody that lives with a chronic illness that I’ve met and come across can live with bouts of anxiety and depression, myself included.” *–39 F, S6509727*
	Cultivate predictable routines	“I have rhythm to my week that helps me know when I’ll have time to rest more or sleep in, compared to times I then have to wake up early. … I tend to do best if I have a pretty solid schedule.” *–25 F, S6561773*	Adjust to new normal	“Everyone’s got a different normal in their life, depending what’s baseline. So accepting my baseline and … being appreciative of my baseline.” *–30 M, S0500585*
	Build contingency plans	“I try to plan everything. … Then if I randomly get stuck at a different hotel and I don't know what's right around there, then that's when I'm like, all right … I already have looked up the updated list.” *–38 F, S9033160*	Acknowledge unpredictable and uncontrollable future	“I don’t want to think too far ahead because I don’t want to get any hopes up. I do still have a deathly fear that I will die like my mother at the age of 40. … I feel like every time I think too far ahead, I get let down.” *–27 F, S8556521*
Socially	Prepare for conversations with others	“I don’t want a pity party from anybody. I think that’s helped me master the situation. I know if somebody’s going to talk to me about it, I know exactly what I’m going to say back.” *–27 F, S8556521*	Rise above ignorant remarks	“When somebody talks down to you after you’ve had something like this, even if it’s in a playful way like, ‘Well, you’re too young to have that. You’re too young,’ it’s hard. … I don’t have to lash out … You have to calm yourself down and know that they don’t know the entire situation.” *–27 F, S8556521*
	Choose where to invest energy	“I learned to just say no to most things. … Instead of my default being yes … I just switched my default to no. Then I would take a time to think about it and I would be like, "You know what? Actually, yes, I can do that." But that preserved what little energy I had.” *–39 F, S2644543*	Accept help from others	“Not being able to drive anywhere has been really hard … I’ve made peace with my parents having to drive me places or my friends that pick me up. I’ve had to make peace without having a car or that sense of independence.” *–24 F, S9633781*

Participants associated increased control with increased knowledge. Seeking out new information helped participants optimize disease management, feel confident communicating with providers, and diminish anxiety driven by uncertainty. Furthermore, over time, participants increasingly relied on experiential knowledge; they learned to “listen to my body” (39 F, S6509727), pay attention to the body’s “signals” (25 F, S6561773), and become “a pro at my own body” (37 F, S4328287). They leveraged this skill not only for their own self-monitoring, but also for self-advocacy with peers and providers who were unfamiliar with IEI or dismissive of their symptoms.

Additionally, participants associated increased control with increased agency. For instance, many participants vigilantly limited exposure to infectious triggers; however, some also said that making deliberate choices about what risks were worth taking enhanced their control. As one participant said, “There's some risks you have to take and some risks you have to avoid…It's a matter of preventing the germs and still living my life, rather than letting the prevention of germs rule my life” (23 M, S3173363).

Several participants maintained control by managing their stress levels through therapy, self-care, and modified relational boundaries. However, some struggled to manage the anxiety of trying to control so many aspects of their illness:

“You have to constantly be monitoring … Then at a certain point, you’re also kind of thinking, ‘Am I just being overly paranoid? Am I overthinking this? Am I just letting my disease take something that wouldn’t be that big of an issue, but because I have my disease, I’m making it a bigger issue than what it actually is?’ I constantly felt that way. You feel kind of ridiculous when you’re reporting a 99.5-degree fever to a doctor. … It’s kind of like a weird balancing act between those two types of thinking. Trying to be on top of it, so that way you don’t wind up in the hospital or to cut something off before it gets worse, to, ‘Am I just overthinking this?’” *–36 M, S6310326*

For this participant, the serious risks associated with common infections made walking the line between appropriate monitoring and hypervigilance feel nearly impossible. Even when symptoms were relatively well-controlled, participants were hesitant to trust they would stay that way. As one participant said: “If it works, it works – but, I mean, will it work forever?” (35 M, S1920177).

Thus, participants were also forced to face the limits of what could be reasonably controlled. For some, this was an ongoing struggle; for others, surrendering the need for control was ultimately liberating. One participant said:

“Each new age, season, that I've entered, I just become more aware of the impact that these illnesses have on my body and on my everyday life. … That's probably where a lot of the anxiety's been coming from, was realizing that lack of control. But at the same time, realizing that because I have such little control, I don't need to carry all of that worry, because it's not helping.” *–28 F, S6531719*

For her, recognizing the impossibility of controlling every risk meant freedom from constantly striving to do so. While she continued to proactively manage her illness, she released herself from blame for every infection and flare-up.

#### Subtheme 2: empowerment through overcoming

For many participants, the greatest burden of IEI was its interruptive impact on their daily lives. They described themselves as “stuck” (37 F, S4328287, 39 F, S0583046), on “pause” (25 F, S6632797), or “hindered from making progress” in life (27 F, S7235706). Often, they used comparative language to contrast their experiences with their healthy peers or their own anticipated narrative for their lives.

“I felt initially very cheated. I was supposed to go to law school. I was going to do the Peace Corps. I had all these plans and things just didn't work out that way for me and I watched my peers moving forward in their careers. … I sort of felt like my life was supposed to be different and it's kind of hard to reconcile myself to the fact that this was the way things are.” *–39 F, S2644543*

As this participant described, the onset of IEI often redirected entire life trajectories. Some participants chose where to live as adults based on access to care. Participants highlighted extra considerations around dating, sex, pregnancy, parenting, travel, beauty, work, school, sports, shopping, and social dynamics. As one said, “I was aware very early on that I wasn’t the same. … It was very evident that they didn’t have to worry about the same things that I did” (36 M, S6310326). She expressed that living with IEI requires living intentionally across many dimensions that others might not give a second thought; this sometimes fostered anxiety and other times cultivated determination.

While IEI often caused permanent life redirection, participants adapted by finding new ways to meet their goals, such as shifting to remote work, or by setting new goals entirely, such as pursuing a new career in advocacy. The determination to overcome barriers was a source of empowerment and self-esteem for these participants.

“Everything that's happened to me has made me who I am and made me this goal-driven, determined person, regardless of anything that goes on. … I can do anything I want with this determination and with the amount of fight, if you will, that these experiences have given to me. I've almost died countless times. I've been on gurneys and tubes hooked up and everything like that. No amount of anxiety at work or no amount of failing a test is ever going to put me back in that situation – so there's nothing that I can't get past.” *–23 M, S3173363*

This participant expressed that his experiences with IEI had altered his life trajectory from what he had expected, but he was proud of the person he had become through that journey.

### Theme 3: Renegotiating community

#### Subtheme 1: unhelpful relationships fail to empathize

Participants shared that their illness and their relationships impacted each other reciprocally: their illness reshaped their relationships, and their relationships influenced their experience of their illness. While living with IEI deepened some relationships, many participants also recounted moments of isolation. As one participant said:

“You feel so alone, so isolated. Nobody is really going to understand unless they have lived it. I think it was over two years before I actually met another patient, and it was another year before I met a patient that had the same exact diagnosis as me. That feeling of isolation and aloneness really can take a toll on a patient’s psyche, emotionally, mentally, spiritually, physically, all of it. It all encompasses all those things.” *–39 F, S6509727*

**Fig. 1:**
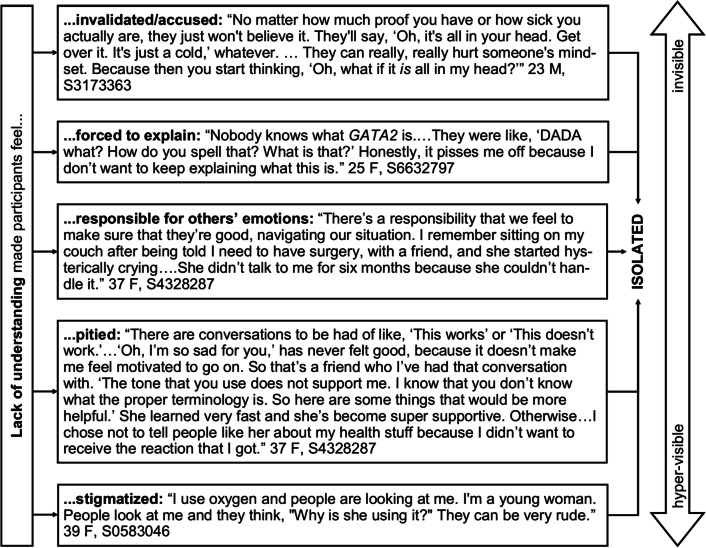
Lack of understanding makes participants feel isolated through invisibility or hyper-visibility

Like many participants, she grounded her experience of isolation in others’ lack of understanding her experience. Participants described five main ways they felt estranged by others’ lack of understanding, which made them feel either invisible or hyper-visible (Fig. 1).

First, participants were hurt by loved ones who refused to accept their illness. These ranged from formal relationships with providers who chose to treat symptoms rather than look for an underlying cause to peers and family members who accused participants of being mistaken, dramatic, or deceitful. Notably, minimizing symptoms and encouraging risk behaviors were the most common reasons participants cited for setting boundaries or ending certain relationships. Second, participants expressed the burden of having to constantly explain their symptoms, correct misconceptions, or justify their choices. Third, participants felt they had to manage others’ emotions in addition to their own, since many people didn’t know how to react to their illness. Fourth, participants struggled with others’ pity or worry. For some, already concerned about burdening others with the demands of their illness, worry reinforced shame. Others felt patronized, victimized, or singled out. Finally, participants described painful experiences of stigma and embarrassment. For many, self-consciousness was particularly acute during adolescence, but for some, it persisted into adulthood, often depending on the visibility of their symptoms.

Participants’ illness, often onset early in life, created a gap between their experiences and their peers’ expectations. Friends and family members who failed to bridge that gap contributed to participants’ negative views of themselves and their illness. Some participants crossed that divide through clear communication about their needs, both physical and emotional, but their ability to do so was dependent on the willingness of the other party to engage in that process. As a result, some relationships were ultimately deemed beyond repair.

#### Subtheme 2: helpful relationships validate and adjust

By contrast, participants felt supported by family members and friends who validated their experiences, adjusted to their needs, and reframed their perspective.

First, when loved ones listened to participants’ accounts of their illness, participants felt supported. Since IEI can be an invisible illness, simply being believed was a crucially validating experience for many participants, especially in contrast to skepticism from others.

Second, participants appreciated those who acknowledged and adapted to their needs, both physical and emotional. For instance, participants said loved ones drove when they were unable, sent cards or text messages to the hospital, and maintained their friendship despite altered rhythms and abilities. As one participant said:

“[My illness] is not something I talk about regularly, so I didn’t even think that they would put two and two together and remember [when the pandemic hit]. So that was kind of nice and heartwarming, that friends and cousins and stuff were like, ‘Hey, what do you need? We can get it if you can’t go out or don’t want to go out.’” *–27 F, S8556521*

Finally, participants valued those who helped them reframe their view of their illness and themselves. Participants often quoted specific conversations that reshaped their perspective: “The person that I am inside – that will always be the same person” (39 F, S0583046); “Nobody was mad at me for not telling them; all they wanted was for me to be happy and healthy” (37 F, S4328287); “You have a chronic illness, but you also have a life to live” (24 F, S9633781). One participant specifically cited her healthcare provider:

“My immunologist, this past year, she kind of said, ‘So what are you doing?’ That’s when I kind of like had a moment of, ‘I actually can do things with the chronic illness.’ Even though I have a central line and stuff and I have to be hooked up to a bookbag and everybody else doesn’t, I can still go to class.” *–39 F, S0583046*

For her, the onset of IEI was so life-shattering that she had to rebuild her identity and understanding of her abilities in the wake of recovery; outside encouragement was an essential catalyst in that process. Another participant similarly highlighted the impact from seeming providers’ seemingly small interventions:

“I've been in a few scenarios where a doctor recognized that I was struggling mentally ... The times when they just took a moment and they were like, ‘I can see that you're really stressed out about this’ – even if I started crying when they said that, I felt better 30 seconds later … For the most part, adults will pull themselves together. It's just that 30 seconds to a minute of being able to let a little bit of it out in the presence of someone who you feel cares about you, at least temporarily. That would go a long way.” *–39 F, S2644543*

When appointments were overwhelming, having the opportunity to voice her anxiety to her provider helped alleviate her distress.

Overall, most participants experienced both helpful and unhelpful relationships; the biggest difference between them was the extent to which loved ones were willing to engage participants around their illness experience rather than either defining them by it or ignoring it altogether. Notably, supportive relationships were marked not necessarily by complete understanding, but simply by validation and encouragement.

#### Subtheme 3: opportunities to give back are meaningful

Importantly, participants also valued opportunities to invest in others. Given the rarity of IEI, many participants found meaning in contributing to research and taking on advocacy roles, either formally or informally.

Participation in research was understood by participants as a way to benefit others in the future, so that “someone doesn’t have to go what I went through, years and years of pain and ups and downs” (40 F, S5228470). Participants’ advocacy activities included providing resources to other patients, educating physicians and fellow patients, volunteering, fundraising, and even changing careers. Communicating their experiences to others was especially meaningful; participants who wrote or spoke in public described it as “cathar[tic]” (25 F, S6561773) and “therapeutic” (37 F, S4328287) in addition to the benefits of educating others.

These activities required time and energy, yet participants said focusing on others gave them a sense of purpose. For example, one said, “If I worry less about myself and more about others, I find that that’s super helpful. It’s really hard to do when you’re not feeling well. …To stay out of your own head and help you stay focused, [helping other people] has been really helpful” (30 M, S0500585).

Genetic diagnosis provided validation and relieved guilt for some, but most participants found meaning apart from causal explanations for their illness. Several participants found more relief in the idea that “there’s just kind of no rhyme or reason to it” (40 F, S5228470) than that “this has to have happened for a reason” (24 F, S9633781). Instead, they emphasized the value of leveraging their experiences to help others, restoring a sense of worth in the wake of their illness.

## Discussion

This study is the first to our knowledge to qualitatively explore psychological adaptation to IEI among affected adults. Participants had to re-evaluate their relationship 1) with themselves, grappling with shame and loss through acceptance and integration; 2) with their circumstances, wrestling for control over fundamentally uncontrollable outcomes; and 3) with others, renegotiating their social ecosystem in the context of new needs. However, the reality is more complex: all three of these relationships overlap and entangle with each other (Ambrosio et al. 2015). Ultimately, participants showed that adaptation to life with IEI is multifaceted, ongoing, and possible.

In many ways, adaptation to IEI required navigating apparent paradoxes. Participants held onto identities beyond their illness even as they allowed themselves to be changed by it. They felt comforted by the shared experience of human illness while feeling isolated by their unique expression of it. They mourned the loss of their expected trajectories while adjusting to new goals. They had to invest significant energy toward controlling their symptoms while simultaneously accepting that those efforts were no guarantee of security. They found peace in allowing themselves to rest and rely on others while being empowered by overcoming obstacles with determined persistence. They balanced their unique experiential knowledge with a need for support and validation from others. They emphasized making meaning *in* their illness rather than *of* it.

If the process of adaptation involves finding a livable balancing point along each of these spectra, every change in illness manifestations or life circumstances adds new weight to either end, forcing recalibration to a new equilibrium. As participants lived with their illness over the course of many years, they constantly readjusted to new challenges. Other rare disease populations have similarly described adaptation as a “wave-like experience” with “dual and shifting natures” (Loesken et al. 2023). Our findings agree with others demonstrating that adaptation is an iterative process that continues throughout the lifespan, with many ups and downs (Ambrosio et al. 2015; Baptist et al. 2024; Paterson 2001).

However, while adaptation has no arrival, progress is still possible. Despite the emergence of new challenges, all participants described psychological adaptation and personal growth over the years since their diagnosis, demonstrating the resilience demanded and developed by life with rare disease.

### Implications for theory

These data add nuance to existing theories of adaptation, highlighting distinctives of rare and chronic disease communities. In accordance with Taylor’s theory (Taylor 1983), our participants affirmed that meaning making and mastery were core concepts. However, participants experienced meaning largely through personal growth and helping others, rather than identifying a causal attribution for their illness. Participants also emphasized that asserting control was balanced by accepting a lack of control, a novel addition to prior frameworks (Setzer et al. 2023; Taylor 1983; Ambrosio et al. 2015). In contrast to Taylor’s participants who compared themselves to cancer patients worse off than themselves, our participants tended to compare themselves to their healthy peers. This distinction may be one area where the divide between rare and common disease becomes relevant. Future research should explore practice implications of these differences across groups and explore the overlap of these factors in other rare and chronic disease communities.

Additionally, participants shared that support was largely experienced through validation as predicted by the social-cognitive processing model (Lepore 2001). While social support literature has emphasized the detriments of isolation, our participants emphasized the impact of negative social interactions as well. These findings align with literature suggesting that relational strain has been historically understudied but should be considered alongside isolation when discussing social support (Hogan et al. 2002). Future studies should further explore the complex social impacts of rare, chronic disease over the life course and identify opportunities for potential interventions.

### Implications for practice

The psychological burden of illness can be as complex and debilitating as the physical burden. Our participants suggested that providers can facilitate psychological adaptation in three key ways: 1) asking, 2) suggesting, and 3) helping.

First, simply asking about a person’s coping can itself facilitate coping. Questions can prompt patients to process emotions, identify opportunities for change, and reflect on their own strengths. Medical providers may ask one or two broad but intentional questions, like “How have you been coping with all of this?” or offer a simple reflection, like “I know this process has been really hard on you.” Providers specifically focused on facilitating adaptation, such as genetic counselors and therapists, may draw on the questions proposed in Box 1 for longer conversations, as well as established therapeutic interventions to facilitate meaning making, enhance perceived control, and identify sources of support (Biesecker and Erby 2008; Helm 2015). As participant S2644543 emphasized, even when these questions bring strong emotions to the surface, most patients are grateful to express these emotions and continue with the clinical encounter.

Box 1 Suggested questions to elicit and facilitate patients’ adaptation processes

**Table:** 

• How have you maintained a sense of self outside your illness? • What would you say to someone else in a similar situation? • Are there ways you feel like you’ve changed, positively or negatively, through this experience? • How has the way you deal with this experience changed over different seasons of life? • Where do you struggle to feel a sense of control? • What do you do when something is beyond your control? • How have your goals changed in the wake of your illness? How do you feel about those changes? • How has your illness impacted your relationships? • What relationships have been helpful or unhelpful to you in adjusting to your illness? • What do you wish more people understood about your experience? • What gives your life meaning as you deal with all of this?	

Second, after listening to a patient’s experience, providers may suggest alternate perspectives, drawing on principles of narrative therapy and strengths-based interventions (MacLeod et al. 2021; Padesky and Mooney 2012; Dane et al. 2024). This does *not* mean finding a “silver lining” or “looking on the bright side.” Rather, this means reminding patients that they are more than their diagnosis, like the immunologist who asked participant S0583046 about life beyond her illness. Providers may be able to reframe a despairing narrative to highlight a patient’s strengths amid their challenging circumstances: “I can see that this has taken a heavy toll on you, and continuing to engage in care while you pursue your goals really shows your resilience and determination.”

Third, while adaptation is not a problem to be solved but a process to engage with, providers can offer practical help. For instance, rather than merely recommending therapy, clinicians can provide specific referrals. Doctors can enhance control by explaining concepts in patient-friendly language. Providers can offer opportunities for meaning and connection by referring patients to advocacy groups. Ideally, these interventions would be tailored to a patient’s specific needs and struggles.

### Limitations

Overall, this study is a novel exploration of the process of adaptation to IEI over the life course. However, these findings should be considered in the context of several limitations. First, there is possible selection bias as all participants voluntarily engaged in a relatively lengthy interview. Although our interviews achieved meaning saturation, it is possible that non-participants may have unique approaches to adaptation. Additionally, the eligible population was drawn from a larger NIH research protocol which may not be representative of all cases of IEI; it is possible this group is enriched for more complex or undiagnosed cases. Finally, the racial and ethnic background of our cohort is over-representative of white, non-Hispanic participants as compared to the broader United States population.

## Conclusion and future directions

Adaptation is a constantly evolving process that requires repeatedly confronting both practical and emotional questions. In alignment with prior studies, meaning, control, and support were key to this process (Taylor 1983; Biesecker and Erby 2008; Similuk et al. 2016; Lepore 2001); this work added nuance to all three concepts through eliciting psychological narratives of integrating illness into life, accepting lack of control, and making meaning beyond causal attributions. Further research is needed to understand the strength and direction of relationships among the factors contributing to adaptation, specific needs of patients across the life course, and optimal interventions to address those needs.

## Supplementary Information

Below is the link to the electronic supplementary material.

Supplementary file1 (PDF 177 KB)

## Data Availability

The interview data generated for this study are not publicly available in order to maintain participant confidentiality. However, de-identified excerpts may be requested for review by contacting the corresponding author.
