# Supplementary material for: “I am sick, but that’s not all that I am”: patient perspectives on psychological adaptation over time to inborn errors of immunity
Source: J Community Genet. 2025 Jan 6;16(2):117–30. doi: 10.1007/s12687-024-00758-z (PMC11979054; doi:10.1007/s12687-024-00758-z)
Supplement: Supplementary file 1 — Supplementary file1 (PDF 177 KB) [file 12687_2024_758_MOESM1_ESM.pdf]

Title: "I am sick, but that's not all that I am": patient perspectives on psychological adaptation to inborn errors of immunity

Authors: Breanna J. Beers[1,2], Hannah R. Davidson[2,3,4], Katie L. Lewis[1], Michael R. Setzer[1,5], Magdalena A. Walkiewcz[1], Morgan N. Similuk[1]

[1]Centralized Sequencing Program, National Institute of Allergy and Infectious Diseases (NIAID), Bethesda, MD, USA.
[2]Department of Health, Behavior, and Society, Johns Hopkins Bloomberg School of Public Health, Baltimore, MD, USA.
[3]Telomere Center at Johns Hopkins, Johns Hopkins University School of Medicine, Baltimore, MD, USA.
[4]Department of Genetic Medicine, Johns Hopkins University School of Medicine, Baltimore, MD, USA.
[5]Department of Medical and Clinical Psychology, Uniformed Services University of the Health Sciences, Bethesda, MD, USA.

Corresponding author: Morgan Similuk, morgan.similuk@nih.gov

Journal: *Journal of Community Genetics*

INTERVIEW GUIDE

To begin with, I'd like to spend just a few minutes up front to hear about your medical diagnosis and what brought you to this point. I've reviewed your medical chart, but I would like to have you share what you think is the most important health context for our conversation from your own perspective.

[Prompt if needed]

- What is the name of your illness or what do you call your illness?
- What age did your symptoms begin and how do they affect you now?
- Does anyone else in your family has a similar illness?

Next, I'm going to move into the main part of the interview, which is learning more about how you have learned to live with your illness. Tell me about your assessment of how you manage your illness, both practically and emotionally, in your life <u>right now</u>.

What has the process been like for you to learn to live with this illness over time?

[Prompt if needed]

- Were there key events in that process?
- Are there important attitudes that you've developed along the way?
- What relationships have been helpful or unhelpful to you in this process?

I'd like to explore this a bit further, in a specific direction. We think about adaptation as having at least two key processes that unfold over time for each individual:

The first is meaning making. With this term, we mean making meaning of your experiences to come to working sense of *what* is happening with your illness, *how* it came to happen in the way it did, and fundamentally, *why* this happened in your life.

- Can you describe the process of meaning making of your illness?
- How has this changed over time for you, if it has?

The second process is gaining mastery. By mastery we mean accumulating a sense of competence with what can be a fundamentally complicated and uncertain process

- Can you describe what this process of gaining this sense of competence has been in your life, if you feel that term applies?
    - How has this changed over time for you, if it has?

From our experience in talking to hundreds of individuals and families in our NIH clinic and what's in the literature, the *context* of what else is happening in one's life and, specifically, where one is in their life course can be important in how individuals make meaning of their illness and gain this sense of mastery. By 'life course,' we mean phases of life such as childhood, adolescence, adult- and later-adulthood. Some phases of life tend to be relatively stable (like middle adulthood) while other phases tend to be have a lot of transition and rapid developmental change (like adolescence and early adulthood).

1. With this in mind: How would you describe your current life phase? What is most relevant for you about this time in your life regarding how dealing with your illness affects you?

   [Prompt if needed]
   - Your context and priorities for this stage of your life?
   - Your internal or external resources?
   - Your sense of social norms?

2. Would you say you have lived with your illness during different periods of your life? If yes, what were those prior life stages like for you in comparison to now? Can you share any of your thoughts on what you imagine the future may be like?

3. As somewhat of an aside, we are working on a separate study on better understanding the personal experiences, attitudes, and needs around reproductive decision-making in individuals with immune disorders. Do you feel like this is relevant to you in the past or now? If so, can you provide some initial comments and we'll circle back to talk about this topic more specifically next summer?

We started this project last summer with an online survey. You may remember completing this for us. In that survey we found that almost 12% or about 1 in 8 of our participants reported experiencing symptoms of <u>severe</u> anxiety or depression in the weeks prior to completing our survey. An even bigger proportion reported mild to moderate symptoms of depression or anxiety, which can still really impact someone's life. This, along with a lot of other data, tells us that having to navigate anxiety and depression when living with a chronic illness is a COMMON experience. Simply, we want to hear more on this topic from your perspective.

1. Have you experienced periods of anxiety or depression that stand out to you? What has this been like?

   [Prompt, if needed]
   - How did you come to recognize that this may be a problem for you?
   - For you, personally, what about these experiences have been most challenging?
   - What, if anything, has been helpful?

We may be interested in contacting some of our interview participants to clarify what they have said or to review our preliminary results with them to gather any further insights.

1. Would you be willing to be re-contacted as part of this process?

2. Do you have any questions or comments for me? Is there anything I haven't asked you about that you'd like to share?

3. Any specific feedback about this process?

That concludes our prepared questions for you. Thank you again for your time. This type of personal, in-depth input from individuals like you is so important to our team, our program and our field.
